# Determinants of social support among people living with HIV in Nigeria–a multicenter cross-sectional study

**DOI:** 10.3389/fpubh.2023.1120192

**Published:** 2023-06-15

**Authors:** Oluwatobi E. Babalola, Oluwaseun A. Badru, Luchuo E. Bain, Oluwafemi Adeagbo

**Affiliations:** ^1^Lagos State Primary Healthcare, Lagos, Nigeria; ^2^Institute of Human Virology, Usmanu Danfodiyo University Teaching Hospital, Sokoto, Nigeria; ^3^International Development Research Centre (IDRC), Ottawa, ON, Canada; ^4^Department of Psychology, Faculty of Humanities, University of Johannesburg, Johannesburg, South Africa; ^5^Department of Community and Behavioral Health, College of Public Health, The University of Iowa, Iowa City, IA, United States; ^6^Department of Sociology, University of Johannesburg, Johannesburg, South Africa

**Keywords:** HIV, ART, social support, family support, friend support, Nigeria

## Abstract

**Background:**

More than 38.4 million people were living with HIV worldwide in 2021. Sub-Saharan Africa bears two-thirds of the burden, with Nigeria having nearly two million people living with HIV (PLWH). Social support from social networks such as family and friends improve the quality of life, and reduces enacted and perceived stigma, but social support for PLWH remains suboptimal in Nigeria. This study aimed to assess the prevalence of social support and associated factors among PLWH in Nigeria and to test whether stigma reduces types of social support.

**Methods:**

This cross-sectional study was conducted in Lagos State, Nigeria, between the months of June and July 2021. A total of 400 PLWH were surveyed across six health facilities providing antiretroviral therapy. Social support (family, friends, and significant others) and stigma were measured with the Multidimensional Scale of Perceived Social Support and Berger’s HIV Stigma Scale, respectively. Binary logistic regression was used to identify determinants of social support.

**Results:**

More than half (50.3%) of the respondents had adequate social support overall. The prevalence of family, friends, and significant others support was 54.3, 50.5, and 54.8%, respectively. Stigma (Adjusted Odds Ratio [AOR]: 0.945; 95% Confidence Interval [CI]: 0.905–0.987) was negatively associated with adequate friend support. Female gender (AOR: 6.411; 95% CI: 1.089–37.742), higher income (AOR: 42.461; 95% CI: 1.452–1241.448), and seropositive disclosure (AOR: 0.028; 95% CI: 0.001–0.719) were associated with adequate significant others support. Stigma (AOR:0.932; 95% CI: 0.883–0.983) was negatively associated with adequate support overall. Our findings corroborate the social support theory, as stigma reduces the chance of receiving social support.

**Conclusion:**

PLWH that enjoy support from families or friends were less likely to be affected by HIV-related stigma. More support is needed by PLWH from family, friends, and significant others to improve the quality of life and reduce stigma among PLWH in Lagos State.

## Introduction

More than 38.4 million people were living with HIV worldwide in 2021 (1). One of the most affected regions is sub-Saharan Africa (SSA), with about two-thirds of the global burden of HIV (2). The countries with the highest burden of HIV in SSA are South Africa, Ethiopia, and Nigeria (3). Nigeria, the most populous country in Africa, has about 1.9 million people living with HIV (PLWH) in 2021 (1).

Current estimates suggest an increase in HIV disclosure in SSA (4, 5). HIV disclosure has been linked to increased social support for PLWH (6). Social support involves relationships and interactions within the relationships that could enhance health and well-being (7). The impact of adequate social support on PLWH includes improved quality of life and overall physical and mental well-being (8). PLWH sometimes, after disclosing their seropositive status, receive support from their social networks, such as family, friends, and colleagues, which positively impacts their quality of life and psychosocial well-being (3, 9). The prevalence of overall support for PLWH is suboptimal and varies in the literature. For instance, in India, the prevalence of strong overall social support was 43.1% (9), 38.6% in Ethiopia (10), and 9.6% in Nigeria (11). The variation in prevalence may be due to geographical and cultural differences (12).

Several factors have been associated with social support among PLWH in the literature, including sociodemographic characteristics such as age, gender, ethnicity, religion, marital status, education, employment, income, medical history such as adherence to antiretroviral therapy (ART), duration of diagnosis, duration on ART, the status of spouse or partner, and partner use of ART (3, 9, 11, 13–15).

There is little knowledge of various types of social support (such as family, friends, and significant others) among PLWH in Nigeria, particularly support from significant others. In the context of this study, significant others (e.g., religious leader and a respected colleague) are those a person feels close to beyond their family and friends (16, 17). Although other studies in SSA have attempted to include other types of support, they often do not treat these support types separately but rather combine them (3, 13, 18, 19). Many of the studies in Nigeria have focused on the overall support of PLWH. For instance, Sule et al. (11) assessed factors associated with overall support, while Folasire et al. (17) assessed overall support and friend support only. Our argument here is that factors associated with support from friends may differ from overall support or even support from family, as shown by Folasire et al. (17). Here, we went one step further by considering social support subtypes separately. We aimed to: (1) assess the prevalence of and factors associated with family support, friend support, support from significant others, and overall support; (2) assess whether increased social support will reduce stigma among PLWH.

## Theoretical framework

The social support theory by Cullen (20) was adopted and tested in this study. It posits that the likelihood of crime and delinquency is more likely to reduce with adequate support (20). Recently, the social support theory was used to test the impact of HIV status disclosure on social support and stigma among PLWH in Uganda (21). Social support has several dimensions, including emotional, perceived, instrumental, and informational support, and can be classified based on the source of support, such as support from significant others, friends, family, and the community (22). There is an associated relief when PLWH disclose their seropositive status, but it can predispose them to stigma (21). Therefore, we tested the impact of stigma on social support as the secondary objective and primarily assessed the determinants of social support.

## Methods

### Study area, study design, setting, and sampling technique

The study was conducted in Lagos State, Nigeria, which has a projected population of 19 million, 37 Local Council Development Areas and 20 local government areas (LGA), including Etio-Osa LGA [Population (23)]. The state has 26 General Hospitals and 256 Public Healthcare Centres, some of which offer ART services [Lagos (24)].

The descriptive cross-sectional study design was adopted in this study. The methodology adopted has been published elsewhere (16). In brief, the Finite formula with a 54.35% prevalence of low support in an earlier study conducted in Dublin (14), 5% margin of error, 95% confidence interval, and sampling frame of 4,212 (active PLWH as of April 2021 across all six facilities). The initial sample size was 350, and 10% was added to cater for non-response and increase study power, which gave a sample of 385 (increased to 400).

### Data collection techniques

Multistage sampling was used to select the respondents between June and July 2021. First, one LGA (Eti-Osa LGA) was selected randomly. Second, six health facilities (Ikate Primary Health Centre [PHC], Ajah PHC, Badore PHC, Iru PHC, St. Kizito hospital, and Police hospital Falomo) were selected with simple random sampling, and the sample size was proportionately allocated to the facilities. Finally, PLWH were recruited from the health facilities using systematic random sampling. Two research assistants administered the questionnaire in each facility because the literacy of the participants could not be ascertained.

## Measures

### Dependent variable

#### Social support

Social support was assessed with the Multidimensional Scale of Perceived Social Support tool (MSPSS) with 12 items. The tool has three subscales - family, friends, and significant others. Each subscale has four items measured on a 7-point Likert scale (1 = Very Strongly Disagree, 2 = Strongly Disagree, 3 = Mildly Disagree, 4 = Neutral, 5 = Mildly Agree, 6 = Strongly Agree, 7 = Very Strongly Agree) (14). Some of the questions in the MSPSS tool include: “*My family really tries to help me*,” “*I can count on my friends when things go wrong*,” and “*There is a special person who is around when I am in need*.” The subscales - family, friends, and significant others - were treated separately and in tandem to assess the impact of social support on PLWH (25). The score ranged from 12 to 84. As previously done in earlier studies, the mean of the score was used to dichotomize each support type and the overall support. A score above the mean was classified as adequate support (coded as 1), and scores below the mean were considered inadequate support (coded as 0) (13, 26). In the present study, the Cronbach α for family, friend, and significant others, and overall support was 0.957, 0.914, 0.908, and 0.910, respectively. These Cronbach α scores vary from strong to excellent (27).

### Independent variables

#### Stigma

Stigma was assessed with Berger’s HIV Stigma Scale. The scale is a 40-item scale measured on a 4-point Likert scale (strongly disagree, disagree, agree, and strongly agree). Some of the questions include: “*Telling someone I have HIV is risky*” and “*People with HIV are treated like outcasts*.” The scale is further categorized into four domains: personalized stigma, disclosure concern, negative self-image, and concerns with public attitudes (28). The overall score ranged from 40 to 160 (items 8 and 21 were reversed). A score between 40 and 80 was categorized as “Low stigma”, a score between 81 and 120 was categorized as “Moderate stigma”, while a score between 121 and 160 was categorized as “High stigma” (28). The scale has a Cronbach α of 0.910 (29). The Cronbach α was 0.920 in the present study, which suggests a strong reliability score (27).

### Adherence to ART

Adherence to ART was measured with self-report. Participants on first-line treatment that reported missing more than one dose of ART within the last 30 days were considered non-adherent to ART; participants on second-line treatment that missed more than four (4) doses of ART within the last 30 days were considered non-adherent to ART. Put differently, participants who missed >5% of ART within a month were considered non-adherent to ART (30, 31).

### Medical history and covariates

The medical history included duration of diagnosis and duration on ART (1–5, 6–10, >10 years) and disclosure of status (Yes, No). Also, spouse HIV status and use of ARV, if positive, were considered. The covariates include eight sociodemographic characteristics: age (18–27, 28–37, >37 years), gender (Male or Female), marital status (Single, Married, Cohabiting, Separated, Divorced, or Widow/er), ethnicity (Yoruba, Igbo, Hausa, or Others), religion (Christianity, Islam, or Others), the highest level of education (None, Primary, Secondary, or Tertiary), occupation (Self-employed, Civil Servant, Unemployed, Student, Others, or Pensioner), and monthly income (< **N** 30000, **N** 30001- **N** 50000, **N** 50001- **N** 100000, or > **N** 100000).

### Data analysis

The SPSS version 26 was used to analyze all data, and the level of statistical significance was set at 5%. Frequency was used to describe categorical variables, while the mean or median was used to describe normal and skewed data, respectively. Where appropriate, Pearson’s Chi-Square or Fisher’s exact test was used to test for the association between the independent variables and social supports. All the variables were considered for the regression analysis. Before regression analysis, multicollinearity was assessed with Variance Inflation Factor (VIF), and VIF <10 was accepted as a non-correlation between the explanatory variables (3). We found evidence of multicollinearity between the duration of diagnosis and the duration on ART. Duration on ART was dropped as it is less common in the literature than diagnosis duration. Also, we found a high correlation and collinearity between the domains of Berger’s HIV Stigma scale and the overall stigma score; therefore, we used the overall stigma score rather than scores from the individual domains. Binary logistic regression analysis was performed to identify factors that predict each of the social support. All analyses were conducted at 95% confidence interval (CI).

### Ethical considerations

Ethical clearance for the study was obtained from the Health Research and Ethics Committee of Lagos University Teaching Hospital (ADM/DSCST/HREC/APP/4400). Also, approval was sought from the Medical Director of the health facilities in Eti-Osa LGA. The interviewer explained in detail the study’s purpose, the benefit of the study, and the risks involved. Written informed consent was obtained from the participants before the interview. Participants were allowed to withdraw from the study at any time without penalty, and the privacy of the participants was ensured during and after the interview. No incentive was given for participation.

## Results

### Sociodemographic, medical history, and social support

Of the 400 questionnaires, 396 were fit for analysis, giving a response rate of 99%. The median age of the respondents was 32 (18–68) years, while the majority were females (59.8%). About 7 in 10 (72.3%) were Christians, and many were single (44.2%). Less than half (47.2%) had secondary school education, while about one-third had tertiary-level education. More than half were self-employed (52.5%), and 16.2% were civil servants (Table 1). Regarding medical history, 8 in 10 were diagnosed with HIV and commenced ART within the last 5 years; however, only 65.5% adhered to ART medications. Seven in 10 respondents had disclosed their seropositive status, and only 20.7% had experienced a high level of enacted stigma. Among those who were married/cohabiting, 4 in 10 (41.2%) of the spouses were living with HIV, and half (50.9%) of these spouses were on ART (Table 2). More than half (50.3%) of the respondents had adequate support overall. Specifically, 54.3, 50.5, and 54.8% of the respondents had adequate family support, friend support, and support from significant others, respectively (Table 3).

**Table 1:** Sociodemographic characteristics (*n* = 396).

Variables	Frequency	Percentage
Age (years)		
18–27	104	26.3
28–37	165	41.7
> 37	127	32.0
Median Age (IQR)	32	18–68
Gender		
Female	237	59.8
Male	159	40.2
Ethnicity		
Yoruba	142	35.9
Igbo	124	31.3
Hausa	37	9,3
Others	93	23.5
Religion (N = 372)		
Christianity	269	72.3
Islam	100	26.9
Others	3	0.8
Marital status		
Single	175	44.2
Married	152	38.4
Cohabiting	19	4.8
Widow/er	28	7.0
Separated	13	3.3
Divorced	9	2.3
Level of education		
None	10	2.6
Primary	52	13.1
Secondary	187	47.2
Tertiary	147	37.1
Employment status		
Self-Employed	208	52.5
Civil Servant	64	16.2
Unemployed	50	12.6
Student	45	11.4
Others	23	5.8
Pensioner	6	1.5
Monthly Income (N) (N = 345)		
< N30,000	117	33.9
30,001 – 50,000	76	22.0
50,001 – 100,000	103	29.9
> 100,000	49	14.2

N, Naira; IQR, Interquartile range (25th –75th).

**Table 2:** Personal and interpersonal medical history (*n* = 396).

Variables	Frequency	Percentage
Duration of HIV Diagnosis		
1–5 years	328	82.8
6–10 years	39	9.8
>10 years	29	7.4
Median Duration of HIV Diagnosis (IQR)	3	1–20
Duration on ART		
1–5 years	328	82.8
6–10 years	41	10.4
>10 years	27	6.8
Median Duration on ART (IQR)	3	1–20
Adherent to ART		
Yes	247	65.5
No	136	35.5
Disclosure of Status (N = 390)		
Yes	280	71.8
No	110	28.2
Stigma		
Low	33	8.3
Moderate	281	71.0
High	82	20.7
Status of partner (N = 170)		
Positive	70	41.2
Negative	96	56.5
Do not know	4	2.3
Use of ART by partner (N = 114)		
Yes	58	50.9
No	56	49.1

HIV, Human Immunodeficiency Virus; ART, Antiretroviral therapy; IQR, Interquartile range.

**Table 3:** Prevalence of social support (*n* = 396).

Variables	Frequency	Percentage
Family support		
Inadequate	181	45.7
Adequate	215	54.3
Mean family support (SD)	18.3	7.7
Friend support		
Inadequate	196	49.5
Adequate	200	50.5
Mean friend support (SD)	14.3	6.1
Significant others support		
Inadequate	179	45.2
Adequate	217	54.8
Mean significant others support (SD)	19.4	6.4
Overall social support		
Inadequate	197	49.7
Adequate	199	50.3
Mean overall support (SD)	52	15.8

SD, Standard deviation.

### Factors associated with adequate support

In the bivariate analysis, age was significantly associated with family, friends, and overall social support only - PLWH above 37 years had more of this support (see Table 4). Specifically, 66.9, 59.8, and 61.4% of participants older than 37 years had adequate support from family, friends, and overall social support, respectively. Ethnicity was only associated with family support. More than two-thirds (67.7%) of other tribes had adequate family support, half (54.2%) of the Yoruba tribe had adequate support, less than half (49.2%) of the Igbos had adequate family support, and more than a third (37.8%) of the Hausa group had adequate family support.

**Table 4:** Association between sociodemographic, medical history and strong social supports.

Variables	Adequate family support			Adequate friend support			Adequate others support			Adequate overall support		
	No (%)	Yes (%)	*p*-value	No (%)	Yes (%)	*p*-value	No (%)	Yes (%)	*p*-value	No (%)	Yes (%)	*p*-value
Age (years)												
18–27	60 (57.7)	44 (42.3)		58 (55.8)	46 (44.2)		47 (45.2)	57 (54.8)		63 (60.6)	41 (39.4)	**0.003**
28–37	79 (47.9)	86 (52.1)	**0.001**	87 (52.7)	78 (47.3)	**0.034**	79 (47.9)	86 (52.1)	0.579	85 (51.5)		80 (48.5)
> 37	42 (33.1)	85 (66.9)		51 (40.2)	76 (59.8)		53 (41.7)	74 (58.3)		49 (38.6)		78 (61.4)
Gender												
Female	114 (48.1)	123 (51.9)	0.243	114 (48.1)	123 (51.9)	0.498	101 (42.6)	136 (57.4)	0.207	118 (49.8)	119 (50.2)	0.984
Male	67 (42.1)	92 (57.9)		82 (51.6)	77 (48.4)		78 (49.1)	81 (50.9)		79 (49.7)		80 (50.3)	
Ethnicity												
Yoruba	65 (45.8)	77 (54.2)		71 (50.0)	71 (50.0)		61 (43.0)	81 (57.0)		67 (47.2)	75 (52.8)	0.150
Igbo	63 (50.8)	61 (49.2)	**0.007**	59 (47.6)	65 (52.4)	0.606	53 (42.7)	71 (57.3)	0.300	61 (49.2)		63 (50.8)
Hausa	23 (62.2)	14 (37.8)		22 (59.5)	15 (40.5)		22 (59.5)	15 (40.5)		25 (67.6)		12 (32.4)
Others	30 (32.3)	63 (67.7)		44 (47.3)	49 (52.7)		43 (46.2)	50 (53.8)		44 (47.3)		49 (52.7)
Religion												
Christianity	116 (43.1)	153 (56.9)	0.313	130 (48.3)	139 (51.7)	0.648	113 (42.0)	156 (58.0)	0.169	127 (47.2)	142 (52.8)	0.246
Islam	49 (49.0)	51 (51.0)		51 (51.0)	49 (49.0)		50 (50.0)	50 (50.0)		54 (54.0)	46 (46.0)	
Marital status												
Single	93 (53.1)	82 (46.9)		104 (59.4)	71 (40.6)		88 (50.3)	87 (49.7)		109 (62.3)	66 (37.7)	**<0.001**
Married	63 (36.8)	108 (63.2)	**<0.001**	67 (39.2)	104 (60.8)	**0.001**	62 (36.3)	109 (63.7)	**0.014**	61 (35.7)	110 (64.3)	
Separated/Divorced	16 (72.7)	6 (27.3)		14 (63.6)	8 (36.4)		13 (59.1)	9 (40.9)		17 (77.3)	5 (22.7)	
Widow/er	9 (32.1)	19 (67.9)		11 (39.3)	17 (60.7)		16 (57.1)	12 (42.9)		10 (35.7)	18 (64.3)	
Level of Education												
None	4 (40.0)	6 (60.0)		6 (60.0)	4 (40.0)		6 (60.0)	4 (40.0)		7 (70.0)	3 (30.0)	**<0.001F**
Primary	35 (67.3)	17 (32.7)	**0.023F**	33 (63.5)	19 (36.5)	0.104F	30 (57.7)	22 (42.3)	0.177F	40 (76.9)	12 (23.1)	
Secondary	82 (43.9)	105 (56.1)		92 (49.2)	95 (50.8)		79 (42.2)	108 (57.8)		86 (46.0)	101 (54.0)	
Tertiary	60 (40.8)	87 (59.2)		65 (44.2)	82 (55.8)		64 (43.5)	83 (56.5)		64 (43.5)	83 (56.5)	
Employment												
Unemployed	38 (48.1)	41 (51.9)		32 (40.5)	47 (59.5)		35 (44.3)	44 (55.7)		41 (51.9)	38 (48.1)	0.081
Civil servant	30 (46.9)	34 (53.1)	0.068	28 (43.8)	36 (56.3)	**0.001**	31 (48.4)	33 (51.6)	0.712	31 (48.4)	33 (51.6)	
Self employed	85 (40.9)	123 (59.1)		102 (49.0)	106 (51.0)		96 (46.2)	112 (53.8)		95 (45.7)	113 (54.3)	
Students	28 (62.2)	17 (37.8)		34 (75.6)	11 (24.4)		17 (37.8)	28 (62.2)		30 (66.7)	15 (33.3)	
Monthly Income (N)												
< N30,000	60 (51.3)	57 (48.7)		70 (59.8)	47 (40.2)		58 (49.6)	59 (50.4)		68 (58.1)	49 (41.9)	**<0.001**
30,001 – 50,000	44 (57.9)	32 (42.1)	**<0.001**	38 (50.0)	38 (50.0)	**0.003**	46 (60.5)	30 (39.5)	**0.006**	48 (63.2)	28 (36.8)	
50,001 – 100,000	30 (29.1)	73 (70.9)		48 (46.6)	55 (53.4)		39 (37.9)	64 (62.1)		36 (35.0)	67 (65.0)	
> 100,000	14 (28.6)	35 (71.4)		14 (28.6)	35 (71.4)		17 (34.7)	32 (65.3)		12 (24.5)	37 (75.5)	
Duration of HIV diagnosis												
1–5 years	163 (49.7)	165 (50.3)	**0.002**	165 (50.3)	163 (49.7)	0.307	154 (47.0)	174 (53.0)	0.247	172 (52.4)	156 (47.6)	**0.033**
6–10 years	11 (28.2)	28 (71.8)		15 (38.5)	24 (61.5)		13 (33.3)	26 (66.7)		12 (30.8)	27 (69.2)	
>10 years	7 (24.1)	22 (75.9)		16 (55.2)	13 (44.8)		12 (41.4)	17 (54.8)		13 (44.8)	16 (55.2)	
Duration on ART												
1–5 years	163 (49.7)	165 (50.3)	**0.002**	165 (50.3)	163 (49.7)	0.320	154 (47.0)	174 (53.0)	0.180	172 (52.4)	156 (47.6)	**0.020**
6–10 years	11 (26.8)	30 (73.2)		16 (39.0)	25 (61.0)		13 (31.7)	28 (68.3)		12 (29.3)	29 (70.7)	
>10 years	7 (25.9)	20 (74.1)		15 (55.6)	12 (44.4)		12 (44.4)	15 (55.6)		13 (48.1)	14 (51.9)	
Adherent to ART												
Yes	123 (49.8)	124 (50.2)	**0.030**	120 (48.6)	127 (51.4)	0.992	116 (47.0)	131 (53.0)	0.276	134 (54.3)	113 (45.7)	**0.014**
No	52 (38.2)	84 (61.8)		66 (48.5)	70 (51.5)		56 (41.2)	80 (58.8)		56 (41.2)	80 (58.8)	
Disclosure of Status												
Yes	78 (70.9)	32 (29.1)	**<0.001**	75 (68.2)	35 (31.8)	**<0.001**	62 (56.4)	48 (43.6)	**0.005**	84 (76.4)	26 (23.6)	**<0.001**
No	99 (35.4)	181 (64.6)		117 (41.8)	163 (58.2)		114 (40.7)	166 (59.3)		109 (38.9)	171 (61.1)	
Stigma												
Low	12 (36.4)	21 (63.6)	0.076	14 (42.4)	19 (57.6)	0.157	11 (33.3)	22 (66.7)	**0.046**	13 (39.4)	20 (60.6)	**0.002**
Moderate	123 (43.8)	158 (56.2)		134 (47.7)	147 (52.3)		122 (43.4)	159 (56.6)		129 (45.9)	152 (54.1)	
High	46 (56.1)	36 (43.9)		48 (58.5)	34 (41.5)		46 (56.1)	36 (43.9)		55 (67.1)	27 (32.9)	
Status of partner												
Positive	20 (28.6)	50 (71.4)	**0.046**	24 (34.3)	46 (65.7)	0.104	29 (41.4)	41 (58.6)	0.100	22 (31.4)	48 (68.6)	0.178
Negative/Do not know	42 (43.8)	54 (56.3)		45 (46.9)	51 (53.1)		28 (29.2)	68 (70.8)		40 (41.7)		56 (58.3)
Use of ART by spouse												
Yes	16 (27.6)	42 (72.4)	**0.019**	16 (27.6)	42 (72.4)	**0.001**	21 (36.2)	37 (63.8)	0.521	16 (27.6)	42 (72.4)	**0.030**
No	30 (48.4)	32 (51.6)		35 (56.5)	27 (43.5)		26 (41.9)	36 (58.1)		29 (46.8)	33 (53.2)	

F: Fisher’s *p*-value; Bolded value of ps are significant at *p*: <0.05

Marital status was significantly associated with all support types. For family support, more than two-thirds (67.9%) had adequate support, which is similar to the 63.2% for married PLWH. A similar pattern was observed for family support and overall social support. Level of education was associated with family and overall support only, where more than half of those with secondary level education (56.1%) and tertiary education (59.2%) got adequate family support, while only a third (32.7%) of those with primary education had adequate family support. A similar pattern was observed with the overall social support. Employment status was significantly associated with only friend support. More than half of the unemployed (59.5%), civil servants (56.3%), and self-employed (51.0%) PLWH had adequate friend support, and nearly a quarter (24.4%) of students living with HIV had adequate support from friends.

Monthly income was significantly associated with all the subtypes and overall social support, and about 7 in 10 PLWH that earned more than N100,000 ($443 to a Naira = $226) had adequate support. We found an association between the duration of HIV diagnosis and family support and overall social support, as increased duration of diagnosis increased family and overall support. PLWH that were adherent to medications had significantly more family and overall support only. Six in 10 (61.8%) PLWH not adherent to ART, and half (50.2%) of those adherent to ART had adequate family support. Similarly, more than half (58.8%) of those not adherent had adequate overall social support, while only 45.7% of those adherent to ART had adequate overall social support.

Interestingly, only 29.1% of those who had disclosed their HIV status and 61.8% of those yet to disclose their seropositive status had adequate family support. This was also true for other social support subtypes and overall social support. Seven in 10 (71.4%) PLWH whose spouses were also living with HIV received adequate family support, while other types of support were not associated with spousal use of ART. Similarly, seven in ten PLWH whose spouses were on ART had adequate support from family and friends. Regarding stigma, support from significant others and overall support were associated with stigma. Specifically, six in 10 PLWH with low support had reported adequate support from significant others and overall support (Table 4).

In the multivariate analysis, all the variables were fitted in a regression model for each support subtype and the overall social support. The independent variables explained 54.8% of the variance in significant others support. None of the variables predicted family support (Table 5). For friend support, stigma reduces the chance of receiving adequate friend support (Adjusted Odds Ratio [AOR]: 0.945; 95% CI: 0.905–0.987). The model for significant others support shows that females were 6.41 times (95% CI: 1.089–37.742) more likely to have adequate support from significant others than males. PLWH earning between N50,001–100,000 were 42.46 (95% CI: 1.452–1241.448) times more likely to get support from significant others than those earning <N30,000 (Table 6). The independent variables explained 50% of the variance in friend support. PLWH that disclosed their seropositive status were less likely to get adequate support from significant others than those that had not disclosed their status (AOR: 0.028; 95% CI: 0.001–0.719). Also, PLWH whose partners are living with HIV were less likely to have adequate support from significant others than those whose partners were not living with HIV (AOR: 0.012; 95% CI: 0.001–0.542).

**Table 5:** Binary logistic regression analysis for family and friend support.

Variable			Adequate family support					Adequate friend support		
	Coefficient	AOR	95%	CI	*p*-value	Coefficient	AOR	95%	CI	*p*-value
Age (years)	0.019	1.019	0.959	1.083	0.539	−0.010	0.990	0.935	1.048	0.731
Gender										
Male (Reference)										
Female	0.534	1.706	0.416	6.985	0.458	1.111	3.037	0.760	12.136	0.116
Ethnicity										
Hausa (Reference)										
Igbo	−0.532	0.587	0.042	8.254	0.693	−1.661	0.190	0.013	2.883	0.231
Yoruba	0.566	1.760	0.190	16.282	0.618	−1.663	0.190	0.021	1.701	0.137
Others	1.199	3.317	0.217	50.684	0.389	−2.186	0.112	0.008	1.627	0.109
Religion										
Islam (Reference)										
Christianity	0.126	1.135	0.248	5.196	0.871	−1.166	0.847	0.185	3.881	0.831
Level of education										
None/Primary (Reference)										
Secondary	1.509	4.524	0.496	41.277	0.181	1.538	4.655	0.512	42.302	0.172
Tertiary	−0.310	0.734	0.051	10.522	0.820	1.121	3.069	0.194	48.502	0.426
Employment										
Unemployed (Reference)										
Employed	0.088	1.092	0.148	8.056	0.931	−2.042	0.130	0.013	1.341	0.087
Monthly Income (N)										
< N30,000 (Reference)										
30,001 – 50,000	−0.745	0.475	0.069	3.261	0.448	1.037	2.822	0.388	20.523	0.306
50,001 – 100,000	1.095	2.988	0.289	30.894	0.358	1.851	6.368	0.557	72.749	0.136
> 100,000	0.993	2.700	0.157	46.344	0.493	1.949	7.018	0.398	123.852	0.183
Duration of HIV Diagnosis	0.238	1.268	0.980	1.641	0.071	−0.072	0.931	0.723	1.199	0.579
Adherent to ART										
No (Reference)										
Yes	−1.164	0.312	0.073	1.334	0.116	0.387	1.472	0.355	6.102	0.594
Disclosure of Status										
No (Reference)										
Yes	−1.718	0.179	0.017	1.911	0.155	3.493	32.899	0.643	1682.052	0.082
Stigma	−0.021	0.980	0.944	1.017	0.285	−0.056	0.945	0.905	0.987	**0.012**
Status of partner										
Negative/Do not know (Reference)										
Positive	−0.615	0.541	0.030	9.677	0.676	−0.734	0.480	0.013	17.954	0.691
Use of ART by spouse										
No (Reference)										
Yes	2.506	12.259	0.589	255.309	0.106	1.433	4.190	0.121	144.681	0.428
Nagelkerke *R*^2^:			0.461					0.500		
Hosmer and Lemeshow *χ*^2^ (*p*-value)			5.114 (0.745)					3.513 (0.898)		

AOR, Adjusted Odds Ratio; HIV, Human Immunodeficiency Virus; ART, Antiretroviral therapy; **N** , Naira; Bolded value of ps are significant at *p*-value: 0.05.

**Table 6:** Binary logistic regression analysis for significant others and overall support.

Variable			Adequate others support					Adequate overall support		
	Coefficient	AOR	95%	CI	*p*-value	Coefficient	AOR	95%	CI	*p*-value
Age (years)	−0.029	0.972	0.907	1.042	0.419	0.001	1.001	0.936	1.070	0.987
Gender										
Male (Reference)										
Female	1.858	6.411	1.089	37.742	**0.040**	1.087	2.965	0.638	13.774	0.165
Ethnicity										
Hausa (Reference)										
Igbo	−0.224	0.799	0.040	15.983	0.883	−0.834	0.434	0.020	9.404	0.595
Yoruba	−0.671	0.511	0.047	5.515	0.580	−1.676	0.187	0.012	2.870	0.229
Others	−1.486	0.226	0.014	3.704	0.297	−2.373	0.093	0.005	1.901	0.123
Religion										
Islam (Reference)										
Christianity	−0.389	0.678	0.123	3.729	0.655	0.142	1.153	0.211	6.314	0.870
Level of Education										
None/Primary (Reference)										
Secondary	0.378	1.459	0.114	18.658	0.772	1.370	3.934	0.370	41.800	0.256
Tertiary	−0.592	0.553	0.024	13.026	0.714	−0.115	0.892	0.048	16.587	0.939
Employment										
Unemployed (Reference)										
Employed	0.036	1.037	0.108	9.950	0.975	−1.542	0.214	0.021	2.198	0.194
Monthly Income (N)										
< N30,000 (Reference)										
30,001 – 50,000	−1.737	0.176	0.021	1.452	0.107	0.137	1.146	0.131	10.042	0.902
50,001 – 100,000	3.749	42.461	1.452	1241.448	**0.030**	3.417	30.463	1.388	668.627	**0.030**
> 100,000	3.196	24.424	0.612	973.959	0.089	3.371	29.106	0.926	915.007	0.055
Duration of HIV Diagnosis	0.266	1.305	0.969	1.757	0.080	0.269	1.309	0.932	1.839	0.121
Adherent to ART										
No (Reference)										
Yes	−0.244	0.783	0.166	3.693	0.758	−0.995	0.370	0.073	1.860	0.227
Disclosure of Status										
No (Reference)										
Yes	−3.588	0.028	0.001	0.719	**0.031**	0.220	1.246	0.107	14.476	0.860
Stigma	−0.003	0.997	0.957	1.039	0.875	−0.070	0.932	0.883	0.983	**0.010**
Status of partner										
Negative/Do not know (Reference)										
Positive	−4.437	0.012	0.001	0.542	**0.023**	−2.532	0.080	0.001	5.121	0.234
Use of ART by spouse										
No (Reference)										
Yes	3.065	21.444	0.738	623.451	0.075	3.225	25.161	0.425	1490.426	0.121
Nagelkerke *R*^2^:			0.548					0.554		
			9.030 (0.340)					6.285 (0.615)		

AOR, Adjusted Odds Ratio; HIV, Human Immunodeficiency Virus; ART, Antiretroviral therapy; **N** , Naira; Bolded value of ps are significant at *p*-value: 0.05.

Only monthly income and stigma predicted adequate overall social support in the overall social support model. The independent variables explained 55.4% of the variance in overall social support. PLWH earning between N50,001–100,000 were 30.5 times (95% CI: 1.388–688.627) more likely to get support from significant others than those earning <N30,000. Those that experienced stigma were less likely to get adequate overall social support (AOR: 0.932; 95% CI: 0.883–0.983).

## Discussion

This study explored the level of family support, friend support, and support from significant others and the associated factors among PLWH in Nigeria. The prevalence of adequate overall social support was 54.3%, corroborating the finding of an earlier study conducted in Jos, Nigeria, where moderate/high support prevalence was 59.6% (11). However, it is higher than the 38.6% strong support reported in Ethiopia (10) and the 48.6% moderate/high social support reported in India (9). Our finding is lower than the 82.4% reported in an earlier study conducted in Ethiopia (32). The plausible explanation for the variation could be different assessment tools, as some of these studies used Oslo Social Support Scale against the MSPSS used in our study. We found that 5 in 10 PLWH have adequate family support and support from significant others, which is not similar to an earlier study conducted in India where they found that 8 in 10 PLWH had sufficient support from family, friends, and others (9). The difference may reflect geographical and cultural variations in support for PLWH (12). Also, the discrepancy may be due to differences in the support classification in both studies.

We found that females had a higher level of support from significant others, which is in contrast to earlier studies conducted in Nigeria (14), and Ethiopia (3); however, two studies are in agreement with our finding (7, 33). The possible explanation given by Li et al. (7) was that females share unpleasant experiences with special persons to reduce psychological pressure, unlike men who choose to adjust without sharing experiences. Also, when women confirm their seropositive status, they are quicker to disclose it to a significant or trustworthy person, which can earn them social support and access to healthcare services (29). This may partly explain our finding that those whose partners were living with HIV were more likely to get significant support, including financial support and emotional resilience (34, 35). In addition, receiving counselling by seroconcordant couples may stimulate improved communication and relationship, and facilitate better coping strategies, according to the World Health Organization (36).

We found that PLWH with higher income experience higher overall social support. This finding comports with earlier studies (7, 11, 13). The explanation for our finding could be that those with higher incomes get more support from family and friends as they may be the family’s breadwinner (7).

PLWH with a lower level of stigma had a higher level of support from friends and overall social support, a finding that echoes the conclusion of studies from sub-Saharan Africa (28, 37) and outside Africa (38). Based on our second objective, our finding supports the social support theory, which posits that higher support reduces stigma among PLWH (21). The plausible explanation could be that a higher level of social support boosts self-esteem, which improves self-worth and social behavior (39), which can buffer the effect of enacted stigma among PLWH (40). Additionally, the fact that most PLWH in our study had disclosed their status may explain why the stigma level was low. According to Oke et al. (28), HIV disclosure is a proxy measurement of stigma and a low level of status disclosure increases stigma.

The major strength of our study was the recruitment of participants from multiple health facilities in Lagos State, which is known as the commercial hub of Nigeria with diverse people and cultures, allowing for a diverse set of participants. Also, this present study examined the prevalence of support from different social networks and the factors that predict adequate support from each social network. However, the limitation of this study is that the findings need to be interpreted with caution because a cross-sectional study cannot establish causality. Longitudinal studies are warranted to understand social support among PLWH in Lagos State and Nigeria at large.

## Conclusion

Our data shows that more than half of PLWH in our study receive adequate support from family, friends, and significant others. Despite not identifying any predictor of family support, adequate support from friends reduces stigma. Further, females, those that earn a higher income, those who have disclosed their status, and those whose spouse was living with HIV get more support from significant others. PLWH needs more support from family, friends, and significant others to improve quality of life and reduce stigma.

## Data availability statement

The original contributions presented in the study are included in the article/supplementary material, further inquiries can be directed to the corresponding author.

## Ethics statement

The studies involving human participants were reviewed and approved by the Health Research and Ethics Committee of Lagos University Teaching Hospital. The patients/participants provided their written informed consent to participate in this study.

## Author contributions

OEB and OAB conceived the concept and designed the methodology, and wrote the manuscript. OEB led data collection. OAB analyzed the data. LB and OA verified data analysis and supervised the entire process. All authors contributed to the article and approved the submitted version.

## Conflict of interest

The authors declare that the research was conducted in the absence of any commercial or financial relationships that could be construed as a potential conflict of interest.

## Publisher’s note

All claims expressed in this article are solely those of the authors and do not necessarily represent those of their affiliated organizations, or those of the publisher, the editors and the reviewers. Any product that may be evaluated in this article, or claim that may be made by its manufacturer, is not guaranteed or endorsed by the publisher.
